# The pathogenic role of interleukin-22 and its receptor during UVB-induced skin inflammation

**DOI:** 10.1371/journal.pone.0178567

**Published:** 2017-05-30

**Authors:** Yejin Kim, Junmyung Lee, Jihoon Kim, Chong Won Choi, Young-Il Hwang, Jae Seung Kang, Wang Jae Lee

**Affiliations:** 1 Laboratory of Vitamin C and Antioxidant Immunology, Department of Anatomy and Cell Biology, Seoul National University College of Medicine, Seoul, Korea; 2 Institute of Allergy and Clinical Immunology, Seoul National University Medical Research Center, Seoul, Korea; 3 Department of Dermatology, Seoul National University Hospital, Seoul, Korea; NYU Langone Medical Center, UNITED STATES

## Abstract

Recent studies show that IL-22, a cytokine produced by activated CD4^+^ T cells and NK cells, plays a pathogenic role in acute and chronic skin diseases. While IL-22 is produced by immune cells, the expression of IL-22Rα, the functional subunit of IL-22R, is mostly restricted to non-hematopoietic cells in organs such as the skin and pancreas. Although it is well known that ultraviolet B (UVB) radiation induces skin inflammation, there have been no reports regarding the effect of UVB on the expression of IL-22Rα. This study investigated IL-22Rα expression and IL-22-mediated proliferation and pro-inflammatory cytokine production by UVB-irradiated keratinocytes. IL-22Rα was increased in HaCaT and primary human keratinocytes after UVB irradiation through the translocation of IL-22Rα from the cytosol to the membrane. This increase in the expression of IL-22Rα was mediated by the PI3K/Akt pathway. Moreover, the suppression of keratinocyte proliferation by UVB irradiation was inhibited by treatment with IL-22. At the same time, IL-22 increased the production of IL-1α, IL-6, and IL-18 in UVB-irradiated HaCaT cells and primary human keratinocytes. Finally, IL-22Rα expression was increased in UVB-irradiated human and mouse skin by immunohistochemistry. The increased expression of IL-22Rα therefore promotes keratinocyte proliferation and pro-inflammatory cytokine production during UVB-induced skin inflammation, suggesting that UVB facilitates skin inflammation by increasing the responsiveness of keratinocytes to IL-22. This study provides a new insight into UVB-induced skin inflammation and the regulation of related inflammatory skin diseases.

## Introduction

IL-22 is a member of the IL-10 cytokine family, and is mainly produced by activated CD4^+^ T cells and NK cells [1, 2]. Its receptor (IL-22R) consists of two subunits, IL-22Rα and IL-10Rβ. The IL-10Rβ subunit is expressed ubiquitously, but the expression of the IL-22Rα subunit is mainly restricted to non-hematopoietic tissues such as the skin, pancreas, intestine, liver, lung, eye, and kidney [3, 4]. There are recent reports that it is also expressed on activated macrophages [5]. Since the biological activity of IL-22 is initiated by binding to IL-22Rα, it is important to track the expression of IL-22Rα in order to understand the actions of IL-22.

IL-22 was recently shown to be associated with acute and chronic skin diseases, and therefore has an important role in inflammatory and wound healing processes in the skin [6–8]. Although IL-22 has anti-inflammatory properties, such as preserving epithelial integrity and promoting wound healing responses, it is also expressed in many chronic inflammatory conditions, such as psoriasis and rheumatoid arthritis, and its upregulation often correlates with disease activity. Recent studies show that IL-22 induces the proliferation of human epidermal keratinocytes obtained from healthy individuals and synoviocytes isolated from psoriatic arthritis, rheumatoid arthritis, and osteoarthritis patients [9–11]. Several studies show that the production of IL-22 from CD4^+^ T cells and NK is induced by IL-6 or IL-23, which are increased during bacterial infection [12–15]. In addition, recent studies show that IL-22 production is increased in inflammatory diseases such as psoriasis and rheumatoid arthritis [16, 17]. Increased IL-22 mediates the progression of inflammatory responses by stimulating the proliferation of keratinocytes and fibroblast-like synoviocytes (FLSs) in each disease [18].

Ultraviolet (UV) radiation is divided into three main categories: UVA (wavelength, 320–400 nm), UVB (280–320 nm), and UVC (180–280 nm) [19]. UVB in particular is closely associated with the development of skin cancer, as it causes DNA damage through multiple mechanisms, including the formation of pyrimidine-pyrimidone (6–4) photoproducts (6–4PP) and cyclobutane pyrimidine dimers (CPDs) [20–23]. Several mechanisms are thought to be involved in UVB-induced skin inflammation [24]. UVB irradiation stimulates the production of inflammatory mediators such as interleukin (IL)-1, IL-6, IL-8, IL-10, and tumor necrosis factor (TNF)-α in keratinocytes, leading to the functional alteration of immune cells in the skin [25, 26]. Activation of the mitogen-activated protein kinases (MAPKs), including the extracellular signal-regulated kinases (ERKs), the c-Jun N-terminal kinases (JNKs), and p38 MAPK, is also associated with UVB-induced skin inflammation [27, 28]. We also recently reported that MAPK signaling cascades are involved in the production of pro-inflammatory cytokines such as IL-1α, IL-1β, IL-6, and IL-18 in the human keratinocyte cell line HaCaT [29]. Nevertheless, more research is necessary to fully understand how UVB induces inflammatory responses in the skin. Moreover, the expression of IL-22 and its receptor, and their role during UVB-induced skin inflammation, are still poorly understood.

The expression of IL-22Rα is also increased in an infectious environment. IFN-α, which is expressed during viral infections, upregulates IL-22α on keratinocytes. In this context, IL-22 inhibits the differentiation of keratinocytes and increases the thickness of the epidermis, thereby mediating innate immune defense against viral infection [30, 31]. In addition, the expression of IL-22Rα is significantly increased in the epidermis of patients with psoriasis [32, 33]. Therefore, this study aims to investigate how the expression of IL-22α in keratinocytes is regulated during a skin inflammatory response induced by UVB, as well as the effects of IL-22 on the proliferation and the pro-inflammatory cytokine production of UVB-irradiated keratinocytes. Our finding suggests that IL-22 is involved in the pathogenesis of both acute and chronic skin diseases; however, the specific mechanisms involved have yet to be elucidated.

## Materials and methods

### Cell culture

HaCaT cells were grown in RPMI 1640 (Welgene, Daegu, Korea) supplemented with 10% heat-inactivated fetal bovine serum (FBS) (ThermoFisher Scientific, Waltham, MA, USA) and antibiotics (100 U/ml penicillin and 100 μg/ml streptomycin; ThermoFisher Scientific) at 37°C in a humidified incubator containing 5% CO_2_. HaCaT cells were provided by Prof. Kyung Chan Park (Department of Dermatology, Bundang Seoul National University Hospital). These cells were initially described by Dr. Norbert E. Fusenig (DKFZ, Heidelberg, Germany) [34].

Primary human keratinocytes were provided by Prof. Jin Ho Chung (Department of Dermatology, Seoul National University Hospital) [35]. Briefly, vascular and adipose tissue from foreskin was removed using scissors. Foreskin was prepared with dispase and incubated with 0.05% trypsin at 37°C for 15 min. To quench the trypsin, DMEM (ATCC, Manassas, VA, USA) containing 10% FBS was added to the keratinocytes. The cells were passed through a sterile sieve and resuspended in a serum-free medium (KBM; BioWhittaker, Heidelberg, Germany) supplemented with 0.1 ng/ml human epidermal growth factor, 0.5 μg/ml hydrocortisone, 5 mg/ml insulin, 7.5 mg/ml bovine pituitary extract, 50 μg/ml gentamicin, 50 ng/ml amphotericin-B, and 0.15 mM calcium). Prior to each experiment, the keratinocyte cultures were determined to be free of contamination with fibroblasts by flow cytometry with an anti-human fibroblast mAb (clone AS02).

### UVB irradiation

#### HaCaT cells and primary human keratinocytes

Cells were grown to 70–80% confluence and washed with PBS prior to UVB irradiation. An XL-1000 UV Crosslinker (Spectronics Corporation, Westbury, NY, USA) was used for UVB irradiation, and a YK-34UV UV light meter (Lutron, Coopersburg, PA, USA) was used for UV dose determination. After UVB irradiation, the cells were replenished with media containing 10% heat-inactivated FBS, and harvested at the time points indicated.

#### Hairless mice (HR-1)

Five-week-old male HR-1 hairless mice (n = 20) were purchased from Samtako Bio Korea (Osan, Korea). They were maintained under specific pathogen-free conditions in the animal facility at the Seoul National University College of Medicine, and all protocols were reviewed and approved by the Ethics Committee of the Seoul National University (Permit number: SNU 100428-3-3). All surgery was performed under sodium pentobarbital anesthesia, and all efforts were made to minimize suffering. The mice were divided into two groups (n = 10 mice/group), as follows: group I, control group without UVB irradiation; group II, irradiated with UVB. The mice were irradiated with 100 J/m^2^ UVB for 13 weeks (5 times/week) on the dorsal skin. After 13 weeks, the dorsal skin tissues, including a 0.5 × 0.5 cm (width × length) section of dermis, were collected from the mice. Next, the tissues were fixed with 4% PFA for immunohistochemistry, as described below.

### Flow cytometry

HaCaT cells (7.5 × 10^5^) were irradiated with 100, 150, or 200 J/m^2^ UVB and cultured for 48 h. The cells were washed with cold PBS, and then resuspended in 1× Annexin V binding buffer (BD Biosciences, San Jose, CA, USA) at a concentration of 2 × 10^6^ cells/ml. One hundred microliters of the solution was transferred into 5 ml round-bottom tubes and stained with FITC Annexin V (BD Biosciences). After 15 min, 7AAD (BD Biosciences) was added and the cells were analyzed on a FACSCallibur instrument (BD Biosciences). FlowJo software (Tree Star, Inc., Ashland, OR, USA) was used for the data analysis.

### RT-PCR

HaCaT and primary human keratinocytes (1 × 10^6^) were harvested 1, 4, and 6 h after irradiation with 100 J/m^2^ UVB. The primers used for RT-PCR were as follows: 5′-CCCCACTGGGACACTTTCTA-3′ (forward) and 5′-TGGCCCTTTAGGTACTGTGG-3′ (reverse) for *IL22RA* (243 bp), and 5′-CATTGGGAATGGTACCAC-3′ (forward) and 5′-CCAATAATGGTGTCATCCAC-3′ (reverse) for *IL10RB* (291 bp). The density of the bands was analyzed with Image J software (NIH, Bethesda, MD, USA).

### Western blotting

#### IL-22Rα expression

HaCaT cells and primary human keratinocytes (1 × 10^6^) were harvested for 24 or 48 h after 100 J/m^2^ UVB irradiation. The cells were lysed, and proteins were extracted using lysis buffer containing 50 mM Tris-HCl (pH 7.4), 1% NP-40, 0.25% sodium deoxycholate, 150 mM NaCl, 1 mM EDTA, and protease inhibitor cocktails. The blocked membrane was incubated with an IL-22Rα Ab (1:4,000; Abcam, Cambridge, UK) and a β-actin Ab (1:8,000; Sigma-Aldrich, St. Louis, MO, USA) overnight at 4℃. The membrane was incubated with horseradish peroxidase (HRP)-conjugated anti-rabbit IgG (1:15,000; Cell Signaling Technology, Danvers, MA, USA) for IL-22Rα and HRP-conjugated anti-mouse IgG (1:10,000; Cell Signaling Technology) for β-actin.

#### Signaling inhibitors

HaCaT cells (7.5 × 10^5^) were pre-treated with specific inhibitors of PI3K/Akt (LY294002, 10 μM; Sigma), p38 MAPK (SB203580, 20 μM; Calbiochem/EMD Millipore, Billerica, MA, USA), JNK (SP600125, 20 μM; Sigma-Aldrich), and ERK (PD98059, 20 μM; Sigma) for 1 h. After washing with PBS, the cells were irradiated with UVB (100 J/m^2^) and cultured for another 24 h for the detection of IL-22Rα.

### Confocal microscopy

HaCaT cells (3 × 10^5^) were grown on 1 cm^2^ cover glasses at 37°C in a 5% CO_2_ atmosphere for 12 h. After washing with PBS three times, the cells were irradiated with 100 J/m^2^ UVB and cultured for another 3 or 6 h. Cells were then collected, fixed with 4% paraformaldehyde (PFA), and pre-incubated with 5% goat serum in PBS-T (0.3% Triton X-100 in PBS) for 1 h. A rabbit anti-human IL-22Rα antibody (Ab) (Abcam) was used as the primary Ab, and an Alexa Fluor 633-conjugated anti-rabbit Ab (ThermoFisher Scientific) was used as the secondary Ab.

### Cell proliferation assays

#### Trypan blue dye exclusion assay

HaCaT cells (7.5 × 10^5^) were irradiated with 100, 150, or 200 J/m^2^ UVB, and incubated with media containing 10% heat-inactivated FBS for 24 h. Cell growth was then measured by trypan blue dye exclusion. Triplicate dishes were averaged, and the percentage of cell growth was calculated as follows: % cell growth = (the number of live cells at 24 h after UVB irradiation / the number of live cells at 24 h without UVB irradiation) × 100.

#### CCK-8 assay

HaCaT cells (1 × 10^6^) were irradiated with 100 J/m^2^ UVB, and then re-plated in a 96-well culture plate at 5 × 10^3^ cells/well, in triplicate. After stabilization, the cells were incubated for another 24 h in the presence or absence of recombinant IL-22 (rIL-22) (20 ng/ml) (R&D Systems, Minneapolis, MN, USA). Cell Counting Kit-8 (CCK-8, Dojindo, Maryland, USA) solution was added (10 μL/well), and cell proliferation was measured at 450 nm using a microplate reader. For the STAT3 inhibitor study, HaCaT cells (1 × 10^6^) were irradiated with 100 J/m^2^ UVB, and then re-plated in a 96-well culture plate at 5 × 10^3^ cells/well, in triplicate. The cells were pre-treated with a specific inhibitor of STAT3 (S3I-201, 50 μM; Sigma-Aldrich) for 3 h. After washing with PBS, the cells were treated with rIL-22 (20 ng/ml) and cultured for another 24 h. Cell proliferation was then measured.

### ELISA

HaCaT and primary human keratinocytes (7.5 × 10^5^) were irradiated with 100 J/m^2^ UVB, and cultured for 24 h in the presence or absence of rIL-22 (20 ng/ml). The concentrations of IL-1α, IL-6, and IL-18 in the culture supernatants were measured by ELISA (R&D Systems).

### Experiments with human samples

#### Subjects

The experiments performed in this study followed the tenets of the Declaration of Helsinki and were approved by the Institutional Ethics Committee of Seoul National University Hospital (protocol 1511-037-718). Skin biopsies with or without UVB and peripheral blood mononuclear cells (PBMCs) were obtained from 30 healthy men aged 19 to 85 years after obtaining written informed consent. All subjects participated in the human irradiation experiments below.

#### Skin biopsy

The buttocks of healthy men were irradiated with 10 mJ/cm^2^ UVB. After 24 h, irradiated and non-irradiated buttock skin samples, including a layer of fat (0.8 × 1 × 0.6 cm), were biopsied after anesthesia. Immunochemistry was used to detect the expression of IL-22Rα in these skin samples, as described below.

### Immunohistochemistry

Human and mouse skin samples were embedded in paraffin and sectioned at a 5 μm thickness. After deparaffinization with xylene and hydration with an alcohol series, tissues were incubated with antigen retrieval buffer [100 mM Tris and 5% (w/v) urea, pH 9.5] at 95℃ for 10 min. After washing with PBS, the tissues were blocked with 5% BSA in PBS-T for 1 h. After serum blocking, the tissue sections were incubated with an Ab against IL-22Rα (1:100; Abcam) overnight at 4℃ in a humidified chamber, and then incubated with a goat anti-rabbit HRP conjugate (1:150). Immunodetection was carried out with 3,3'-diaminobenzidine (DAB), and nuclei were counter-stained with hematoxylin and eosin (H&E). After mounting, the tissues were observed by inverted light microscopy (Olympus, Center Valley, PA, USA).

### Isolation of PBMCs

PBMCs were obtained from the blood of healthy individuals by density gradient centrifugation using Ficoll-Paque Plus (GE Healthcare, Pittsburgh, PA, USA) [36]. To induce IL-22 production, PBMCs at 2.5 × 10^6^ cells/ml were stimulated with 5 μg/ml concanavilin A (Con A) and cultured in complete RPMI 1640 media containing 10% heat-inactivated FBS (ThermoFisher Scientific) and antibiotics (100 U/ml penicillin and 100 μg/ml streptomycin; ThermoFisher Scientific) for 48 h. IL-22 levels in the culture supernatant were measured with an IL-22 ELISA kit (BioLegend, San Diego, CA, USA).

### IL-22 bioassay

Culture supernatants from PBMCs stimulated with 5 μg/ml Con A and those from untreated control PBMCs were concentrated 10-fold by ultrafiltration using Ultracel YM-10 (EMD Millipore, Billerica, MA, USA). The concentrated supernatants were filtered using 0.22 μm pore microfilters, and used to treat UVB-irradiated HaCaT cells in a 96-well plate for 24 h. The bioactivity of IL-22 in the culture supernatant against the IL-22Rα expressed by the UVB-irradiated HaCaT cells was measured by CCK-8 proliferation assay, and, in some experiments, 2.5 μg/ml of an anti-human IL-22 neutralizing Ab (R&D Systems) was used.

### Statistical analysis

All values are represented as the means ± standard deviation (SD). Unpaired two-tailed Student’s t-tests were used to compare effects between groups. The statistical analysis was carried out using GraphPad InStat, version 5.01 (GraphPad Software, La Jolla, CA, USA).

## Results

### UVB irradiation increases the expression of IL-22Rα in human keratinocytes

**Fig 1:**
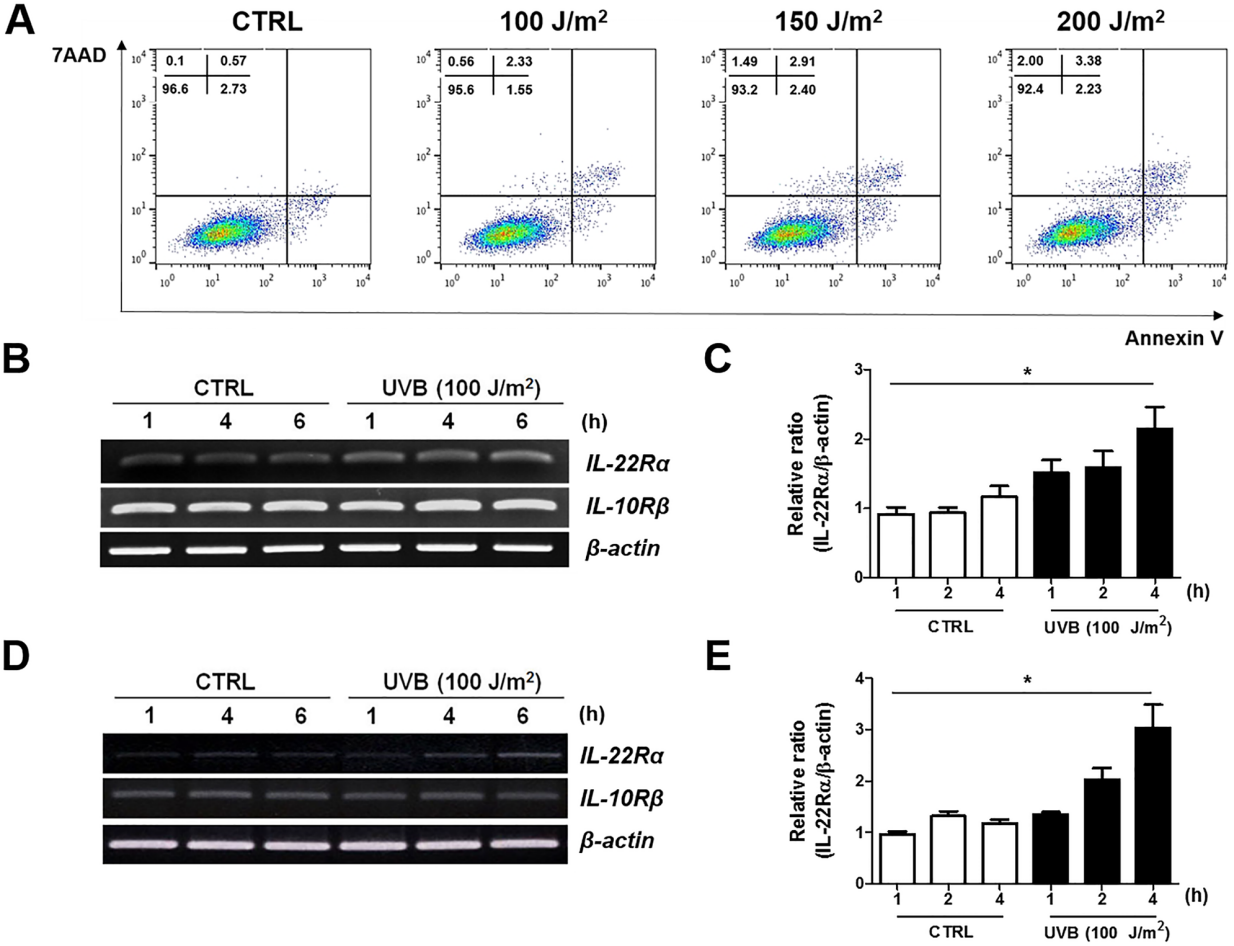
The effect of UVB irradiation on cell viability and IL-22Rα expression. (A) HaCaT cells (7.5 × 10^5^) were irradiated with 100, 150, or 200 J/m^2^ UVB and then cultured for 48 h. The cells were collected and stained with Annexin V-FITC and 7AAD. The effect of UVB on cell viability was measured by flow cytometry. Results are representative of five experiments. (B-D) HaCaT cells (B, C) and human primary keratinocytes (D, E) were collected at 1, 4, and 6 h after irradiation with 100 J/m^2^ UVB, and analyzed by RT-PCR. Results are representative of three experiments. For densitometry analysis, the relative expression of each band was normalized against the expression of β-actin. *p < 0.05.

First, a dose-kinetic study was performed to determine the highest dose of UVB irradiation that would not induce cytotoxicity. Cell viability was decreased at 48 h with 150 and 200 J/m^2^ UVB, but not with 100 J/m^2^ (Fig 1A). Therefore, experiments on IL-22Rα expression in HaCaT cells and primary human keratinocytes were performed using 100 J/m^2^ UVB.

**Fig 2:**
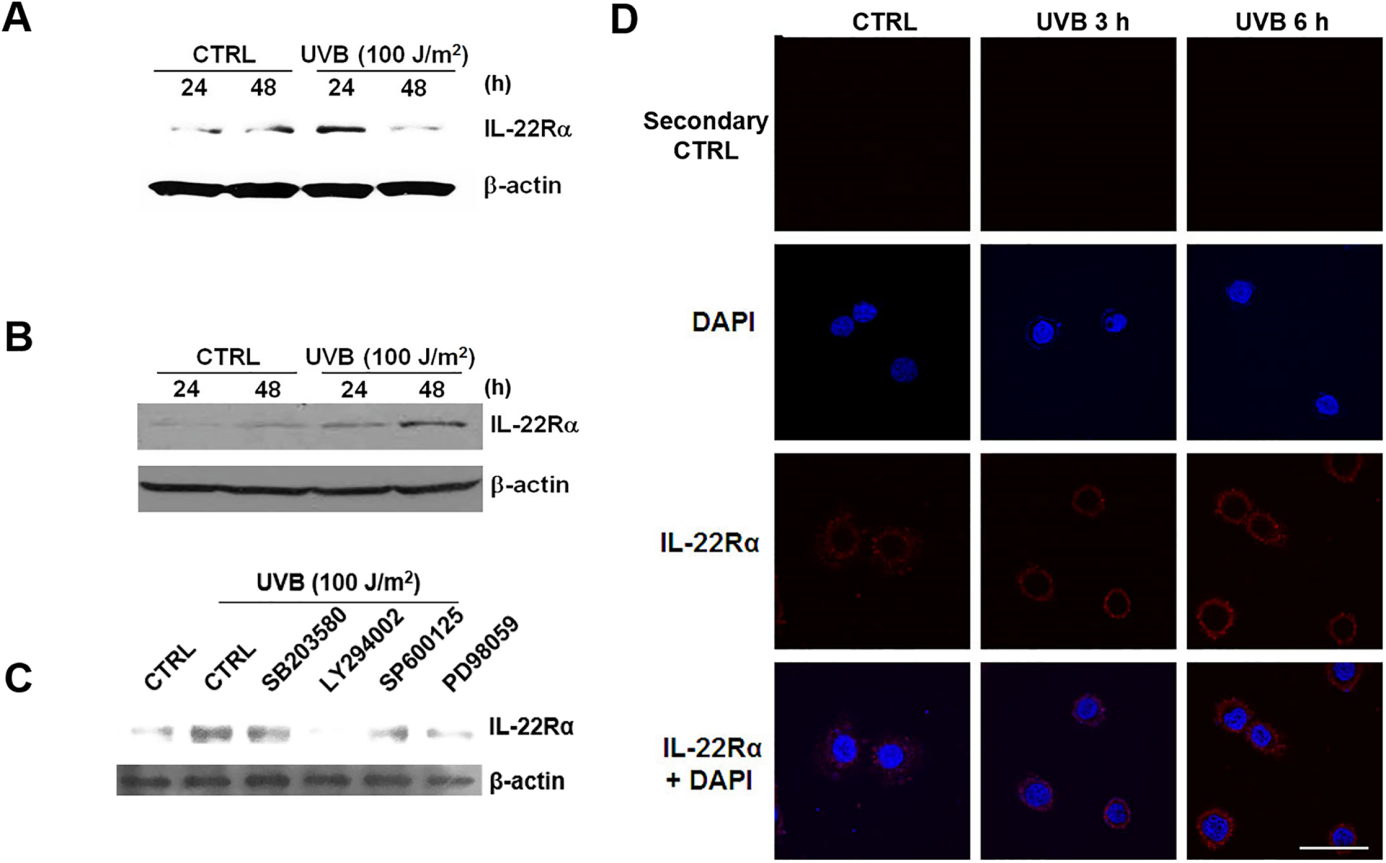
The effect of UVB irradiation on the expression of IL-22Rα and signaling pathway involvement. (A and B) HaCaT cells (A) and human primary keratinocytes (B) were collected 24 and 48 h after being irradiated with UVB. The cells were lysed, and protein was extracted for western blot analysis. (C) HaCaT cells were pre-treated with DMSO (vehicle control), LY294002 (10 μM), SB203580 (20 μM), SP600125 (20 μM), or PD98059 (20 μM) for 1 h prior to irradiation with 100 J/m^2^ UVB. After another 24 h, protein was extracted for western blot analysis. (D) HaCaT cells were collected 3 and 6 h after UVB irradiation and fixed with 4% PFA for confocal microscopy. Results are representative of three independent experiments. Scale bar = 40 μm.

IL-22Rα mRNA expression increased in a time-dependent manner following UVB irradiation, whereas IL-10Rβ, a co-receptor of IL-22Rα to form functional IL-22R, was constitutively expressed (relative IL-22Rα expression in HaCaT cells: control, 1.16 ± 0.16; UVB, 2.15 ± 0.32; *p* = 0.0492) (primary keratinocytes: control, 1.17 ± 0.07; UVB, 3.04 ± 0.45; *p* = 0.0145) (Fig 1B–1E). The expression of IL-22Rα on the surface of HaCaT cells (Fig 2A) and primary keratinocytes (Fig 2B) was confirmed at the protein level by western blot analysis after 24 and 48 h of UVB irradiation. Next, the signaling pathways involved in IL-22Rα expression during UVB irradiation were investigated using HaCaT cells pre-treated with inhibitors of specific signaling pathways: LY294002 (PI3K/Akt), SB203580 (p38 MAPK), SP600125 (JNK), and PD98059 (ERK). Only LY294002, a specific inhibitor of PI3K/Akt, inhibited UVB-induced IL-22Rα expression at the transcriptional (S1A and S1B Fig) and translational levels (Fig 2C). The phosphorylation of Akt in HaCaT cells after 100 J/m^2^ UVB irradiation was confirmed by immunoblotting (S1C Fig). Finally, the induction in IL-22Rα expression on the surface of HaCaT cells by UVB irradiation was examined using confocal microscopy. Interestingly, the surface expression of IL-22Rα increased within 6 h of UVB irradiation (Fig 2D). This finding suggests that UVB irradiation induces IL-22Rα expression on the cell surface by promoting the translocation of IL-22Rα from the cytosol to the cell membrane, rather than through *de novo* synthesis.

### IL-22 rescues the suppressed proliferation of UVB-irradiated HaCaT cells

**Fig 3:**
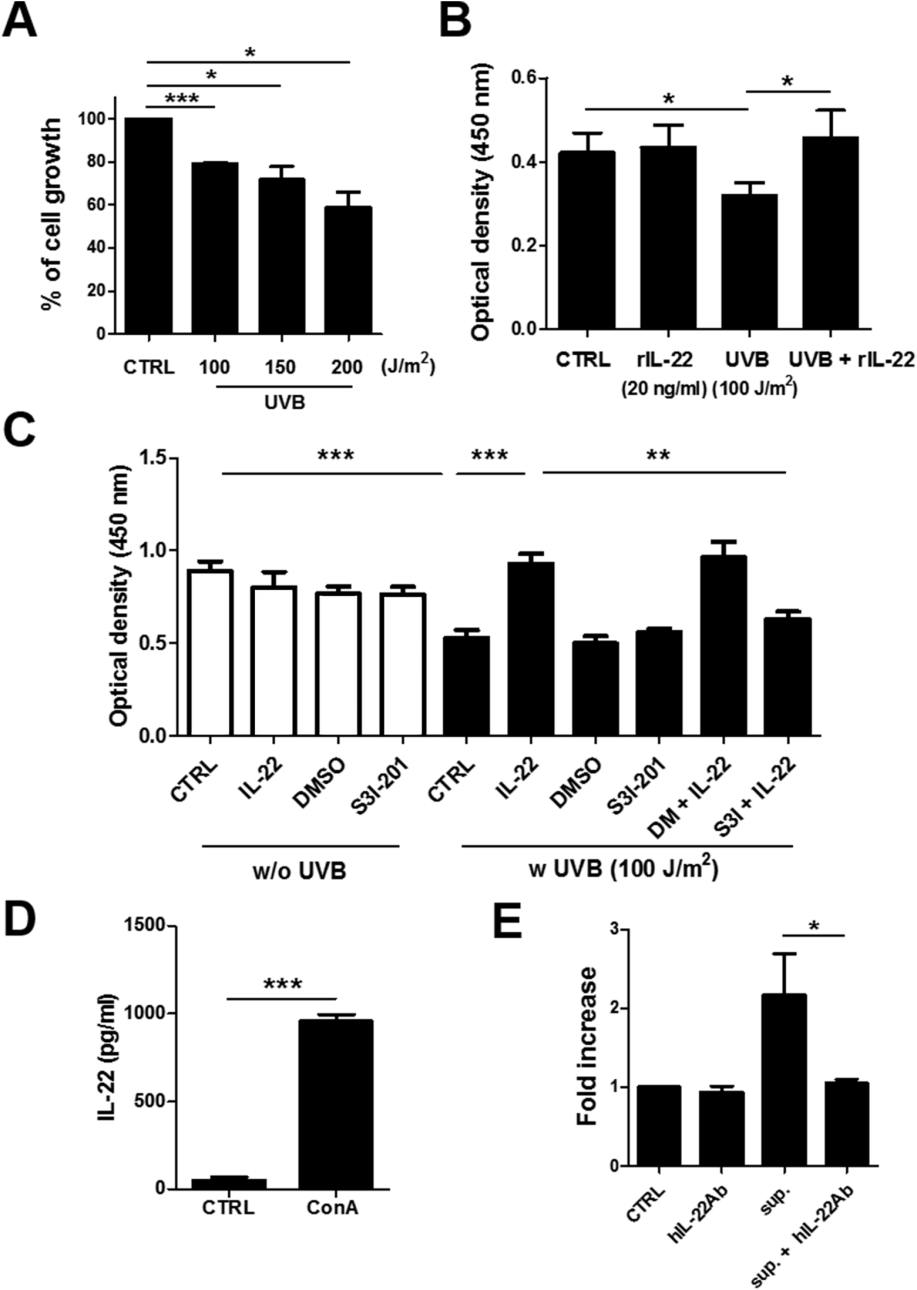
Rescue of the suppressed proliferation of UVB-irradiated HaCaT cells by rIL-22 and IL-22-producing PBMCs. (A) HaCaT cells were irradiated with UVB and then cultured for 24 h. (B) HaCaT cells were cultured in the presence or absence of human rIL-22 (20 ng/ml) for 24 h after UVB irradiation. (C) UVB-irradiated HaCaT cells were pre-treated with S3I-201 for 3 h, and then treated with rIL-22 (20 ng/ml) for another 24 h. (D) PBMCs were cultured in the presence or absence of Con A for 48 h. (E) UVB-irradiated HaCaT cells were treated with culture supernatant from activated PBMCs in the presence of a hIL-22 Ab. Proliferation was normalized to the proliferation of control cells for each experimental group. *p < 0.05, **p < 0.001, ***p < 0.0001.

As shown in Fig 3A, proliferation of HaCaT cells was suppressed by UVB irradiation (100 J/m^2^, 79.05 ± 0.39% cell growth compared with control-treated cells; 150 J/m^2^, 71.61% ± 3.54%; and 200 J/m^2^, 58.70% ± 4.23%; *p* = 0.0003, *p* = 0.0152, and *p* = 0.0103, respectively). As shown in Figs 1 and 2, IL-22Rα expression was increased at both the transcriptional and post-transcriptional level after UVB irradiation. In addition, Cho *et al*. reported that IL-22 was associated with keratinocyte proliferation [32]. Therefore, we investigated the effect of IL-22 treatment on the proliferation of UVB-irradiated HaCaT cells, and found that the UVB-induced suppression of HaCaT cell proliferation was completely restored in the presence of IL-22 (control, 0.42 ± 0.03; rIL-22, 0.43 ± 0.03; UVB, 0.32 ± 0.15; and UVB with rIL-22, 0.46 ± 0.04) (control vs. UVB, *p* = 0.0171; UVB vs. UVB with rIL-22, *p* = 0.0121) (Fig 3B). As shown in Fig 3C, S3I-201, a STAT3-specific inhibitor, inhibited the effect of IL-22 on UVB-irradiated HaCaT cells (control, 0.89 ± 0.05; UVB, 0.53 ± 0.04; rIL-22 with UVB, 0.93 ± 0.06; S3I plus rIL-22 with UVB, 0.63 ± 0.04) (control vs. UVB, *p* = 0.0006; UVB vs. rIL-22 with UVB, *p* = 0.0005; rIL-22 with UVB vs. S3I plus rIL-22 with UVB, *p* = 0.0023). These results demonstrate that activation of STAT3 plays an important role in the IL-22 signaling pathway.

The ability of IL-22 produced by activated immune cells to restore the suppressed proliferation of UVB-irradiated HaCaT cells was investigated next. Increased IL-22 was detected in culture supernatants from Con A-activated PBMCs (control, 52.29 ± 7.63 pg/ml; Con A, 956.3 ± 19.98 pg/ml; *p* < 0.0001) (Fig 3D). The proliferation of HaCaT cells in the presence of these culture supernatants was then examined. Like the results obtained with rIL-22, as shown in Fig 3B, the UVB irradiation-induced suppression of HaCaT cell proliferation was inhibited after treatment with culture supernatants from Con A-activated PBMCs. However, this result was not observed when the IL-22 in the culture supernatant was neutralized with anti-IL-22 neutralizing Ab (supernatant alone, 2.17 ± 0.30; supernatant plus hIL-22 Ab, 1.05 ± 0.03; *p* = 0.0200) (Fig 3E).

### IL-22 increases the production of IL-1α, IL-6, and IL-18 in UVB-irradiated keratinocytes

**Fig 4:**
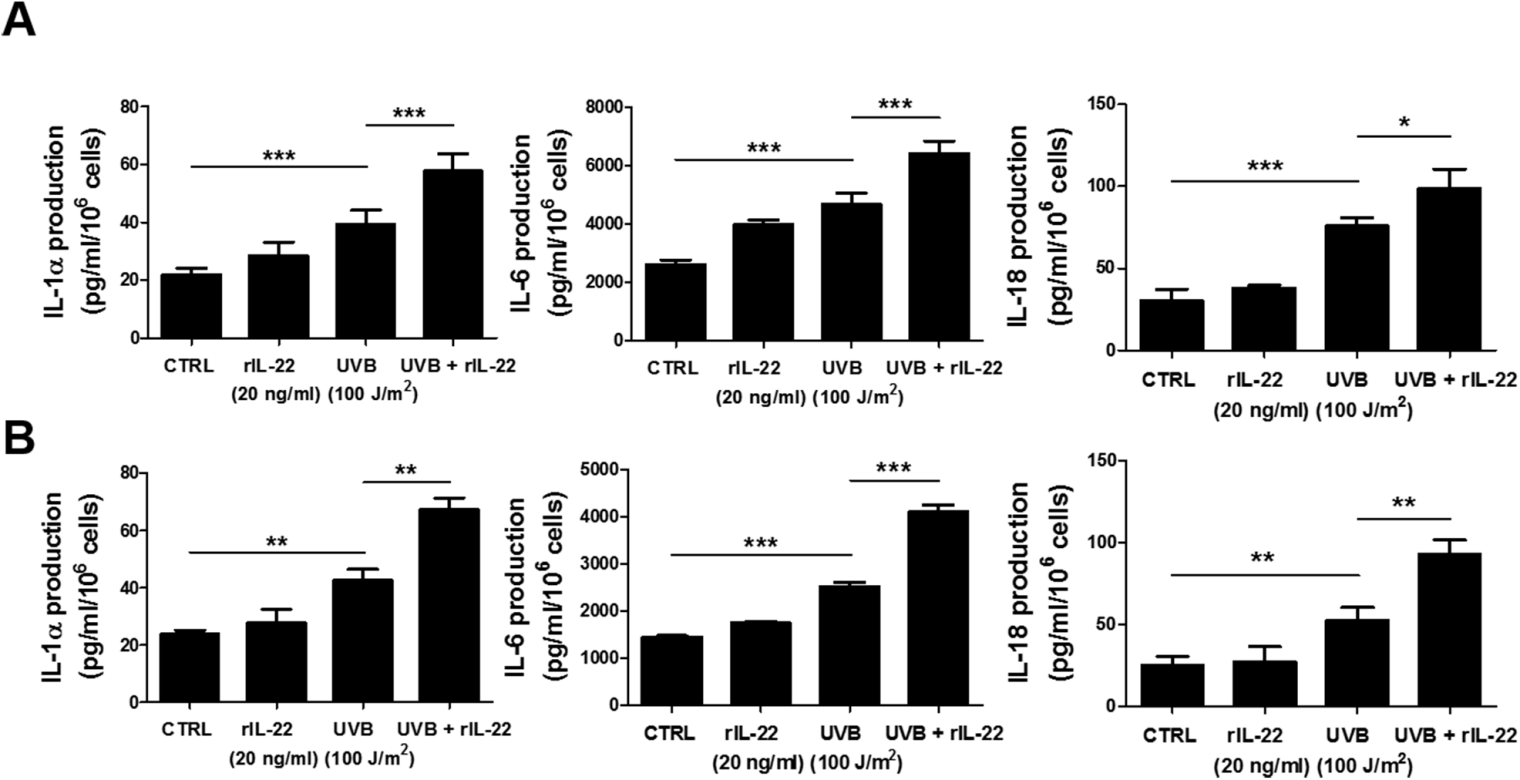
Increased production of IL-1α, IL-6, and IL-18 in UVB-irradiated HaCaT cells and primary human keratinocytes by IL-22. (A and B) HaCaT cells (A) and primary human keratinocytes (B) were irradiated with 100 J/m^2^ UVB and then cultured for 24 h in the absence or presence of rIL-22 (20 ng/ml). Culture supernatants were collected, and the levels of IL-1α, IL-6, and IL-18 were measured by ELISA. Each sample was run in triplicate, and the results are representative of three independent experiments. The data are presented as the means ± SD. *p < 0.05, **p < 0.001, ***p < 0.0001.

To investigate whether IL-22 is involved in the production of inflammatory cytokines by UVB-irradiated keratinocytes, HaCaT cells and primary human keratinocytes were treated with rIL-22, and the secretion of pro-inflammatory cytokines was measured by ELISA. As shown in Fig 4, the production of IL-1α, IL-6, and IL-18 from HaCaT cells and primary human keratinocytes was increased by UVB irradiation, and further increased in response to treatment with rhIL-22. These data suggest that increased IL-22Rα induced by irradiation on HaCaT cells and primary keratinocytes enhances the production of cytokines involved in skin inflammation.

### UVB increases IL-22Rα expression on keratinocytes in mouse and human skin

**Fig 5:**
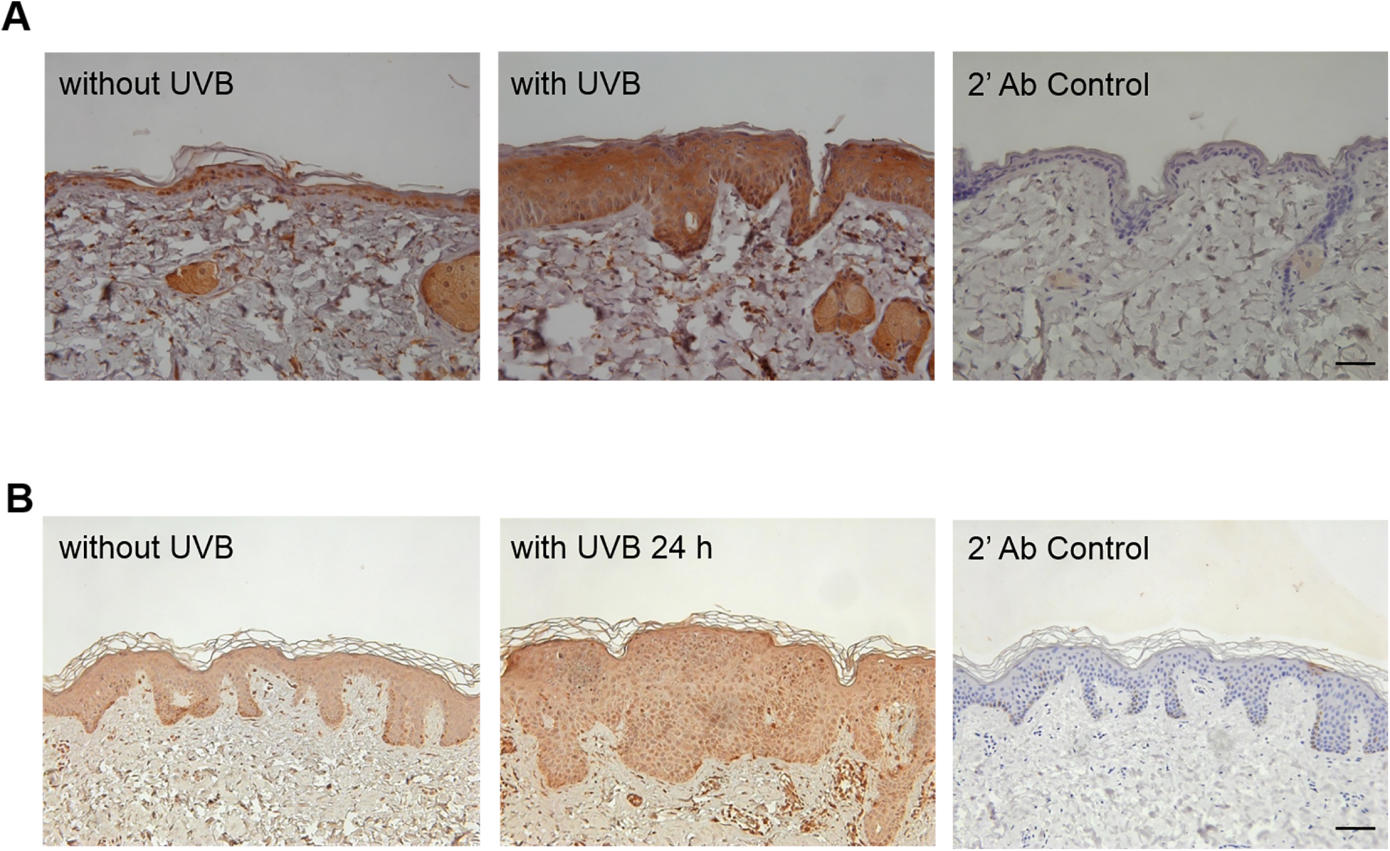
IL-22Rα expression in a representative mouse skin and human skin biopsy. (A) Five-week-old male HR-1 hairless mice (n = 20) were irradiated with 100 J/m^2^ UVB for 13 weeks (5 times/week) on the dorsal skin. (B) The buttocks of healthy men were irradiated with 10 mJ/cm^2^ UVB. After 24 h, skin samples were biopsied from non-irradiated and irradiated areas. Immunochemistry was used to detect the expression of IL-22Rα in these mouse and human skin samples, and hematoxylin and eosin staining was performed to confirm skin inflammation (not shown). Scale bar = 200 μm.

Since the expression of IL-22Rα in HaCaT cells and primary keratinocytes is increased by UVB irradiation (Figs 1 and 2), we examined the effect of UVB irradiation on IL-22Rα expression in mouse and human skin. The induction of skin inflammation in response to UVB irradiation was confirmed histologically in the biopsy samples. The expression of IL-22Rα was increased in skin during an inflammatory response induced by UVB (Fig 5A and 5B). Thus, the induction of skin inflammation by UVB is accompanied by the expression of IL-22Rα in both humans and mice.

## Discussion

UVB is a well-known inducer of skin cancer. Whether IL-22 and IL-22Rα are involved in the development of skin diseases, including inflammation and cancer, has not yet been reported. Studies show that there is a statistically significant difference in the expression of epidermal IL-22Rα between psoriasis and normal skin, as well as between psoriasis and vitiligo skin [16, 37]. In addition, Tohyama *et al*. reported that IFN-α enhances IL-22Rα expression in keratinocytes [30]. However, whether UVB irradiation increases IL-22Rα expression during a skin inflammatory response has not been determined. In this study, we observed the increased expression of IL-22Rα in UVB-irradiated keratinocytes (Figs 1 and 2) and the increased proliferation of UVB-exposed cells upon IL-22 stimulation. IL-22Rα expression in response to UVB irradiation was also observed within inflamed skin in HR-1 mice (Fig 5). Thus, this study is the first to demonstrate that an increase in IL-22 production and IL-22Rα expression may be associated with inflammatory skin disease. Furthermore, the observation that UVB mediates this process may be helpful in the treatment of inflammatory skin diseases caused by UV rays.

UVB induces skin inflammation through the production of inflammatory mediators including LL-37, IL-1, IL-6, and IL-18 in the skin [38]. Th17 cytokine (IL-17 and IL-22) upregulates the expression of CXCL8 and IL-6 in the skin of atopic dermatitis patients and promotes inflammation of the skin [39]. However, it remains unclear whether UVB induces skin inflammation by increasing the expression of IL-22 and its receptor. One of the interesting findings from this study is that UVB increased the production of IL-1α, IL-6, and IL-18 by increasing IL-22 Rα expression (Fig 4). Given that IL-1α and IL-6 are involved in the pathogenesis of skin diseases caused by proliferation of keratinocytes [40–42], IL-22-dependent regulation of UVB-induced IL-1α, IL-6, and IL-18 production may be important in the mechanism of inflammatory skin diseases. Additional studies regarding the effect of UVB-induced IL-22 production and IL-22Rα expression on the production of IL-1α, IL-6, and IL-18 will provide a basis for further understanding the role of IL-22 in the control of inflammatory skin disease. Taken together, our results suggest that UVB-induced skin inflammation is regulated through the modulation of IL-22 production.

There are many recent studies reporting that IL-22 increases keratinocyte proliferation [9, 12, 32]. Here, we showed that the inhibition of keratinocyte proliferation by UVB did not occur in the presence of IL-22 (Fig 3B). Recently, Donetti *et al*. also reported that IL-22 is involved in keratinocyte differentiation [43]. Given that UVB mainly induces an inflammatory reaction in the epidermal layer, it is thought that UVB is unlikely to directly stimulate T cells under the epidermal layer to secrete IL-22. However, interestingly, UVB irradiation directly induced IL-22 production from cultured T cells, although there was no difference between the 100 and 150 J/m^2^ dose levels (control, 15.50 ± 41.15 pg/ml; 200 J/m^2^, 65.05 ± 15.89 pg/ml; *p* = 0.0358) (S2 Fig). Further research is needed to determine whether this phenomenon is actually observed *in vivo*.

The increased expression of IL-22Rα on keratinocytes by UVB demonstrates that IL-22 is involved in the induction and progression of skin inflammation induced by UVB. Interestingly, IL-22Rα expression at the transcriptional level was not changed by exposure to UVB (Fig 1B and 1D), but its surface expression was increased 3 h after UVB irradiation (Fig 2D). This finding suggests that UVB induces the translocation of IL-22Rα from the cytosol to the cell surface. The increased expression of this receptor may promote the inflammation in the skin by increasing the responsiveness of keratinocytes to IL-22. We previously reported that SVCT-1, a specific channel for vitamin C uptake, also migrates from the cytoplasm to the cell surface following UVB irradiation of keratinocytes, although this molecule is functionally different from IL-22R in terms of its anti-inflammatory mechanism [44]. Molecules that migrate to the cell surface relatively quickly after radiation exposure may be useful as diagnostic markers to detect inflammation, and may have utility as therapeutic targets for symptom relief within the context of UVB-induced skin inflammation.

In general, it is known that UVB promotes skin cancer via intracellular DNA damage. Therefore, we investigated the role of IL-22 and IL-22Rα in the induction of skin cancer by analyzing cell cycle changes in keratinocytes exposed to UVB and then treated with IL-22. In addition, even though the data are not presented here, we found that IL-22Rα is also highly expressed on the surface of melanoma cells. While this suggests that IL-22 may also promote the development and progression of melanoma, further investigation is needed. Moreover, the regulation of IL-22 production or IL-22Rα expression might be an effective novel target for skin cancer therapy. Currently, we are developing an Ab that can block IL-22R and inhibit skin inflammation and cancer caused by UVB. Studies are being conducted with this Ab in preventive and therapeutic models of skin inflammation and skin cancer.

In conclusion, this study is the first to report an increase in IL-22Rα expression in keratinocytes after UVB irradiation. The functional consequence of the increased expression of IL-22Rα seems to be increased responsiveness of the keratinocytes to IL-22 stimulation. IL-22 therefore plays a major role in facilitating inflammatory responses in the skin, as it increases the proliferation of UVB-irradiated keratinocytes and the production of inflammatory cytokines. Therefore, this study provides new insight into UVB-induced skin inflammation and the regulation of related inflammatory skin diseases.

## Supporting information

S1 Fig The role of specific signaling pathways in UVB-induced IL-22Rα expression in HaCaT cells. (A) HaCaT cells were pre-treated with DMSO (vehicle control), SB203580 (20 μM), SP600125 (20 μM), PD98059 (20 μM), LY294002 (10 μM), or Bay11-7082 (5 μM) for 1 h and then irradiated with 100 J/m^2^ UVB. After 6 h, total RNA was extracted for RT-PCR analysis with specific primers for *IL22RA*. (B) Densitometry analysis was used to compare the relative expression of IL-22Rα with the expression of β-actin. (C) HaCaT cells were irradiated with 100 J/m^2^ UVB, and then collected at the indicated time for western blot analysis. **p < 0.001.
(TIF)

S2 Fig The production of IL-22 by UVB-irradiated PBMCs. PBMCs isolated from healthy donors were seeded onto inserts (0.4 μm pore size) in a 6-well plate. Then, the cells were irradiated with 100, 150, or 200 J/m^2^ UVB. After 48 h, culture supernatants were collected for the measurement of IL-22 production by ELISA.
(TIF)

S1 File Supplementary materials. (DOCX)

S2 File Supplementary materials. (DOCX)
