# Supplementary figures and images for: The pathogenic role of interleukin-22 and its receptor during UVB-induced skin inflammation
Source: PLoS One. 2017 May 30;12(5):e0178567. doi: 10.1371/journal.pone.0178567 (PMC5448782; doi:10.1371/journal.pone.0178567)

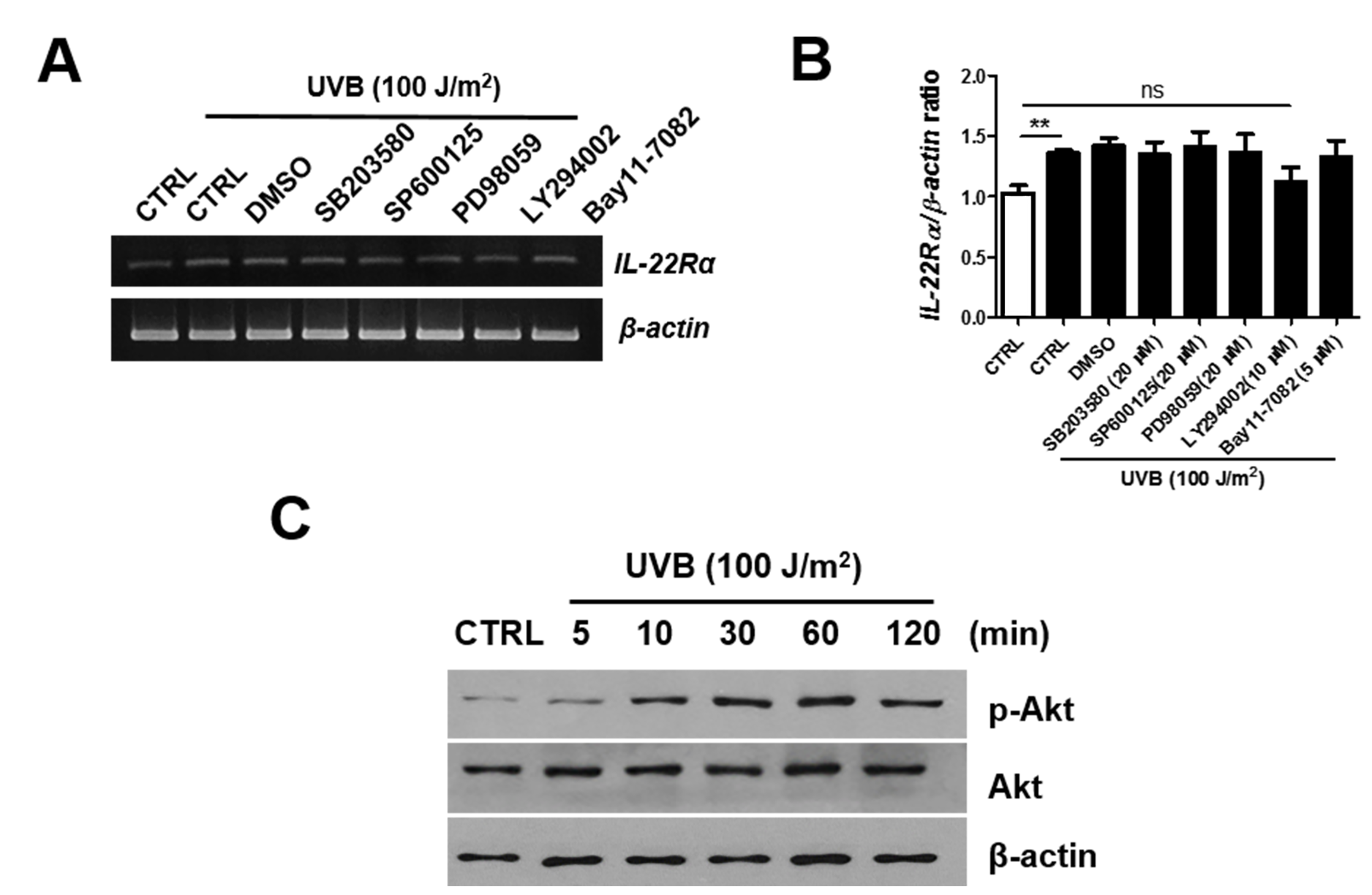

Supplement: S1 Fig — (A) HaCaT cells were pre-treated with DMSO (vehicle control), SB203580 (20 μM), SP600125 (20 μM), PD98059 (20 μM), LY294002 (10 μM), or Bay11-7082 (5 μM) for 1 h and then irradiated with 100 J/m2 UVB. After 6 h, total RNA was extracted for RT-PCR analysis with specific primers for IL22RA. (B) Densitometry analysis was used to compare the relative expression of IL-22Rα with the expression of β-actin. (C) HaCaT cells were irradiated with 100 J/m2 UVB, and then collected at the indicated time for western blot analysis. **p < 0.001. (TIF) [file pone.0178567.s001.tif]

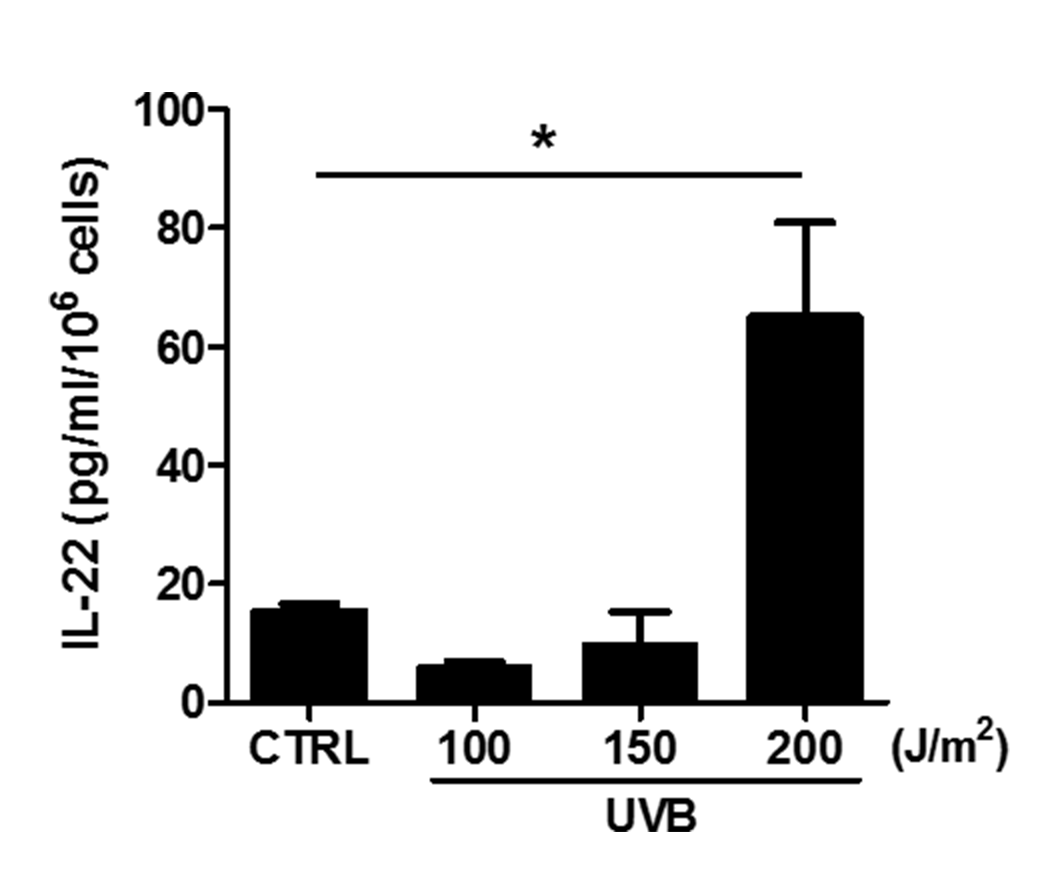

Supplement: S2 Fig — PBMCs isolated from healthy donors were seeded onto inserts (0.4 μm pore size) in a 6-well plate. Then, the cells were irradiated with 100, 150, or 200 J/m2 UVB. After 48 h, culture supernatants were collected for the measurement of IL-22 production by ELISA. (TIF) [file pone.0178567.s002.tif]
