# Supplementary material for: The pathogenic role of interleukin-22 and its receptor during UVB-induced skin inflammation
Source: PLoS One. 2017 May 30;12(5):e0178567. doi: 10.1371/journal.pone.0178567 (PMC5448782; doi:10.1371/journal.pone.0178567)
Supplement: S1 File — (DOCX) [file pone.0178567.s003.docx]

S1 Fig.

**Materials and Methods**

**Examination of signaling pathways required for IL-22Rα expression**

HaCaT cells (7.5 × 10^5^) were pre-treated with specific inhibitors of p38 MAPK (SB203580, 20 μM; EMD Millipore, MA, USA), JNK (SP600125, 20 μM; Sigma-Aldrich), ERK (PD98059, 20 μM; Sigma-Aldrich), PI3K/Akt (LY294002, 10 μM; Sigma-Aldrich), and NF-κB (Bay11-7082, 5 μM; Sigma-Aldrich) for 1 h. After washing with PBS, the cells were irradiated with UVB (100 J/m^2^) and cultured for an additional 6 h.

**S1 Fig. The role of specific signaling pathways in UVB-induced IL-22Rα expression in HaCaT cells.** (A) HaCaT cells were pre-treated with DMSO (vehicle control), SB203580 (20 μM), SP600125 (20 μM), PD98059 (20 μM), LY294002 (10 μM), or Bay11-7082 (5 μM) for 1 h and then irradiated with 100 J/m^2^ UVB. After 6 h, total RNA was extracted for RT-PCR analysis with specific primers for *IL22RA*. (B) Densitometry analysis was used to compare the relative expression of IL-22Rα with the expression of β-actin. (C) HaCaT cells were irradiated with 100 J/m^2^ UVB, and then collected at the indicated time for western blot analysis. ***p* < 0.001.
