# Supplementary material for: The pathogenic role of interleukin-22 and its receptor during UVB-induced skin inflammation
Source: PLoS One. 2017 May 30;12(5):e0178567. doi: 10.1371/journal.pone.0178567 (PMC5448782; doi:10.1371/journal.pone.0178567)
Supplement: S2 File — (DOCX) [file pone.0178567.s004.docx]

S2 Fig.

**Materials and Methods**

**UVB irradiation of PBMCs**

PBMCs isolated from healthy donors were seeded onto an insert with a 0.4 μm pore size at 1 × 10^6^ cells/ml, in a 6-well plate. After centrifugation at 2,000 rpm for 5 min, the cells were irradiated with UVB at 100, 150, or 200 J/m^2^. After 48 h of culture, the supernatants were collected and analyzed for IL-22 content by ELISA.

**S2 Fig. The production of IL-22 by UVB-irradiated PBMCs.** PBMCs isolated from healthy donors were seeded onto inserts (0.4 μm pore size) in a 6-well plate. Then, the cells were irradiated with 100, 150, or 200 J/m^2^ UVB. After 48 h, culture supernatants were collected for the measurement of IL-22 production by ELISA.
